# Predicting multi-label emojis, emotions, and sentiments in code-mixed texts using an emojifying sentiments framework

**DOI:** 10.1038/s41598-024-58944-5

**Published:** 2024-05-28

**Authors:** Gopendra Vikram Singh, Soumitra Ghosh, Mauajama Firdaus, Asif Ekbal, Pushpak Bhattacharyya

**Affiliations:** 1 Department of Computer Science and Engineering, Indian Institute of Technology Patna, Patna, 801103 India; 2 Department of Computer Science and Engineering, Indian Institute of Technology Bombay, Mumbai, 400076 India; 3 University of Alberta, Edmonton, Alberta Canada

**Keywords:** Computer science, Computational science

## Abstract

In the era of social media, the use of emojis and code-mixed language has become essential in online communication. However, selecting the appropriate emoji that matches a particular sentiment or emotion in the code-mixed text can be difficult. This paper presents a novel task of predicting multiple emojis in English-Hindi code-mixed sentences and proposes a new dataset called *SENTIMOJI*, which extends the SemEval 2020 Task 9 SentiMix dataset. Our approach is based on exploiting the relationship between emotion, sentiment, and emojis to build an end-to-end framework. We replace the self-attention sublayers in the transformer encoder with simple linear transformations and use the RMS-layer norm instead of the normal layer norm. Moreover, we employ Gated Linear Unit and Fully Connected layers to predict emojis and identify the emotion and sentiment of a tweet. Our experimental results on the *SENTIMOJI* dataset demonstrate that the proposed multi-task framework outperforms the single-task framework. We also show that emojis are strongly linked to sentiment and emotion and that identifying sentiment and emotion can aid in accurately predicting the most suitable emoji. Our work contributes to the field of natural language processing and can help in the development of more effective tools for sentiment analysis and emotion recognition in code-mixed languages. The codes and data will be available at https://www.iitp.ac.in/~ai-nlp-ml/resources.html#SENTIMOJI to facilitate research.

## Introduction

Emojis have evolved into a ubiquitous component of contemporary communication, offering individuals a means to convey emotions and nuances within their text. The task of predicting emojis has gained paramount significance in the realm of Natural Language Processing (NLP), propelled by the extensive adoption of social media platforms^1–4^. In today’s digital landscape, individuals frequently utilize multiple emojis to articulate their sentiments and emotions, as depicted in Table 1. However, discerning the precise emoji corresponding to a specific emotion or sentiment proves challenging, particularly in code-mixed text where multiple languages coalesce.

Code-mixing is a linguistic phenomenon that occurs frequently in multilingual communities, where speakers tend to switch between two or more languages while communicating^5,6^. Code-mixing introduces a layer of complexity in sentiment and emotion analysis. Social media posts, especially on platforms like Twitter, frequently amalgamate languages, posing challenges in deciphering the emotional and sentiment-laden content. For instance, the following tweet: *“What a wicket!* @*iamamirofficial we love you. Kya kar diya apney?”* (Translated: “What a wicket! @iamamirofficial we love you. What have you done?”) illustrates such code-mixed text, which makes identifying emotions and sentiments more challenging.

The connection between emojis, sentiment, and emotion is intricate and pivotal for understanding the layers of meaning within code-mixed text. Sentiment, expressing one’s opinion as positive, negative, or neutral, and emotion, revealing feelings towards a subject (e.g., angry, joyful), are closely linked with emojis, as affirmed by previous studies^7,8^. To illustrate, consider the tweet “Exploring the haunted house .” Despite the initial excitement suggested by “Exploring the haunted house,” the addition of the disgust puke face emoji indicates a shift in sentiment and emotion. This combination implies that the individual’s experience of exploring the haunted house was unpleasant and elicited feelings of disgust. Therefore, the tweet as a whole conveys a negative sentiment and a sense of repulsion or discomfort towards the haunted house. This scenario further illustrates the close relationship between emojis, sentiment, and emotion, as the choice of emoji can significantly alter the interpretation of the text’s underlying sentiment and emotion.

**Table 1:** Illustrating the presence of multiple emojis in tweets.

Tweets	English Translation	Emojis
I love Narendra Modi ab kya karun wo hein hi ache PM!!!!	I love Narendra Modi what to do, he is a good PM!!!!	,
Mujhe nafrat hai is app se app, its really annoying.	I hate this app its really annoying	,
Aaj kal ki media ghin ati hai they are really bad	These days media I feel disgust they are really bad	

Tasks such as emoji detection, sentiment classification, and emotion recognition are often interdependent and correlated. They perform better when addressed simultaneously^9^. The task of predicting multiple emojis in code-mixed sentences can be a multi-task learning problem exploiting the correlation between emotion, sentiment, and emojis. Identifying the sentiment and emotion of a given tweet can assist in predicting the most appropriate emoji that accurately represents the sentiment and emotion. Similarly, the presence of an emoji in a tweet can aid in identifying the sentiment and emotion, potentially improving the performance of sentiment analysis and emotion recognition models^10,11^.

This paper introduces a novel task that focuses on predicting multiple emojis in code-mixed sentences while concurrently identifying the corresponding emotion and sentiment of the user. Our method utilizes a zero-shot strategy to integrate emotional information into the training process. Our objective is to leverage the interdependence among emotion, sentiment, and emojis and create a comprehensive framework that can accurately detect all three components in code-mixed text. In this work, we enhance the F-Net with RMS-Normalization and introduce Gated Linear Unit (GLU) and Fully Connected (FC) layers for predicting emojis and identifying the emotion and sentiment of a given tweet. To facilitate our research, we introduce the *SENTIMOJI* dataset by extending the benchmark sentiment annotated SentiMix Dataset^12^ of code-mixed tweets with manual annotations for multi-label emojis.

The proposed multi-task framework can help bridge the research gaps in the field of sentiment and emotion analysis. Although several studies have been conducted to predict emojis, sentiment, and emotion separately, there is a lack of research that focuses on predicting them simultaneously in code-mixed sentences. Therefore, our work can advance the field of sentiment and emotion analysis by providing a comprehensive understanding of the relationship between emojis, sentiment, and emotion in code-mixed sentences. Previous studies have examined the correlation between emojis and emotions or sentiment independently, without addressing the complexity of capturing the simultaneous relationship between multiple emojis, sentiment, and emotion in a multi-task framework. The intricacy of human emotions and the use of multiple emojis to express a complete meaning make it essential to accurately comprehend the overall sentiment of a tweet by identifying multiple emojis in it. Hence, predicting the appropriate emojis is crucial for comprehending a tweet’s sentiment. The challenge is further compounded when dealing with code-mixed data, requiring an implicit understanding of the relationship between emotion, sentiment, and emoji to accurately predict the associated emojis. Our work introduces a novel end-to-end multi-task framework that aims to address the challenge of detecting multiple emojis and their corresponding sentiments and emotions in code-mixed data. To the best of our knowledge, this is the first attempt to utilize sentiment and emotion information in a multi-task framework to solve the problem of multi-label emoji prediction in code-mixed data.

### Broad objectives

The primary goal of this study is to develop a robust framework for multi-label emoji prediction in code-mixed text, enriched with sentiment and emotion analysis. Our objectives include:

- Introducing a novel task of predicting multiple emojis in English-Hindi code-mixed sentences while identifying the corresponding emotion and sentiment of the user.
- Proposing a zero-shot strategy to integrate emotional information into the training process, facilitating accurate emotion classification in an unsupervised setting.
- Developing an end-to-end framework that exploits the correlation between emotion, sentiment, and emojis to simultaneously identify the emotion, sentiment, and corresponding emojis in code-mixed text.
- Extending the sentiment annotated SentiMix 2020 dataset with manual annotations for multi-label emojis on 20k code-mixed texts, resulting in the *SENTIMOJI* dataset.
- Demonstrating the effectiveness of the proposed framework by outperforming state-of-the-art models on the *SENTIMOJI* dataset.
- Open-sourcing the code and data to facilitate further research (https://www.iitp.ac.in/~ai-nlp-ml/resources.html#SENTIMOJI).

The structure of our paper is as follows: In the “Related Work” section, we provide a comprehensive review of previous studies related to our research. Next, we introduce our proposed methodology for multitask experiments in the “Methodology” section, which outlines the steps we took to develop our model. We describe the dataset and its annotations in detail in the “About Dataset” section, where we provide information on the corpus used in our experiments. The “Experiments, results, and analysis” section presents the experiments conducted, the obtained results, and our analysis of those results. Lastly, in the “Conclusion” section, we summarize our findings and suggest potential avenues for future research.

## Related work

Predicting emojis has become a crucial task in NLP due to the widespread use of emojis in social media. Therefore, several studies have focused on the analysis of emojis, sentiment, and emotion, which are important areas in the field of NLP.

Cowie and Cornelius^13^ were among the pioneering researchers who explored the significance of emotions in natural language processing (NLP). Subsequent studies have utilized emojis in NLP, including Eisner et al.^14^, who introduced pre-trained emoji2vec embeddings that analyze emoji usage and relationships between different emojis. Other studies, such as Felbo et al.^15^, Zhou et al.^16^, and Jin et al.^2^, developed models to predict emojis in a text, while Barbieri et al.^17^ and Wang et al.^3^ used pre-trained emoji2vec embeddings to create models for single-label and multi-lingual emoji prediction, respectively. Guibon et al.^18^ proposed an emoji recommendation model for instant messaging, while Wijeratne et al.^19^ developed a model for emoji sense disambiguation. In addition, emojis were used by Santhanam et al.^20^ and Hussien et al.^21^ to understand crisis events and classify emotions, respectively. Emojis were also utilized in sentiment analysis by Al-Baaj et al.^22^ and Chen et al.^23^, and by Zhou et al.^16^ for emotional response generation. Moreover, Ma et al.^24^ proposed a transformer-based network for multi-label emoji prediction, which outperformed previous models on benchmark datasets. These studies demonstrate the importance of emojis in NLP and suggest new directions for research in this area. Kada et al.^25^ presents a novel architecture for generating sarcastic sentences enriched with emojis. It leverages valence reversal and semantic incongruity to create sarcastic sentences when words alone may fail to convey sarcasm effectively.

Sentiment and emotion analysis in code-mixed texts has gained increasing interest among researchers in recent years. Various techniques have been proposed to enhance the accuracy of sentiment and emotion detection in code-mixed data. For instance, Chakravarthi et al.^26^ developed a Dravidian code-mixed dataset to recognize sentiment and offensive language in Tamil-English, Kannada-English, and Malayalam-English texts. Research by Mohbey et al.^27^ employed a hybrid approach combining Convolutional Neural Networks (CNNs) and Long Short-Term Memory networks (LSTMs) to effectively analyze sentiment in Monkeypox tweets. This work builds upon the foundations laid by studies in tweet sentiment analysis, incorporating the strengths of both CNNs and LSTMs for improved sentiment classification. Yadav et al.^28^ suggested using pre-trained language models to transfer knowledge from monolingual text to code-mixed text for sentiment analysis. Zhang et al.^29^ represents the the close connection between sentiment and emotion in human feelings, leading to the interdependence of sentiment analysis and emotion recognition in NLP. Addressing conversational context dependency, multi-modal interaction, and multi-task correlation, the proposed M3GAT, a multi-modal, multi-task interactive graph attention network, solves these challenges in a unified framework. In contrast, Wang et al.^30^ proposed a joint factor graph model that accounts for the interactions between different languages and emotions for emotion prediction in code-mixed texts. Wang et al.^31^ introduced a Bilingual Attention Network (BAN) model that utilizes attention mechanisms to aggregate monolingual and bilingual informative words for predicting emotions in code-mixed data. Xiao et al.^32^ introduces a network for multimodal aspect-based sentiment analysis, aligning and fusing information from diverse modalities, addressing the limitation of previous sentiment analysis focused primarily on textual data. Mao et al.^33^ explores how biases in the training data affect the performance of language models in these tasks and provides insights into the limitations and potential mitigations for biased predictions. Existing studies have primarily focused on single-label emoji classification tasks, leaving a gap in understanding the challenges and strategies required for multilabel emoji identification within the complex and varied context of Twitter data^34^ tried to fill this gap. He et al.^35^ proposed a method which leverages meta-learning techniques to improve the efficiency and effectiveness of aspect-based sentiment analysis in scenarios where labeled data is scarce. Mao et al.^36^ introduce a novel gating mechanism to facilitate communication between towers of multi-task learning, enhancing the model’s ability to perform both aspect-based sentiment analysis and sequential metaphor identification concurrently. The study conducted by Meena et al.^37^ introduces a hybrid deep learning approach for detecting sentiment polarities in Monkeypox tweets. Additionally, the research incorporates knowledge graph representation techniques, showcasing an interdisciplinary approach that combines sentiment analysis and knowledge graph methodologies. Recent research has demonstrated the potential of advanced machine-learning techniques in emoji prediction tasks.^38^ study aimed to explore the correlation between emojis, emotions, and sentiment. Studies such as^24^ and^39^ have shown promising results using these techniques. The latter study went further by incorporating multimodal information, including both text and images, for multimodal emoji prediction. Other research has also applied emoji prediction, as seen in^40–44^. For example,^45^ used emojis for irony detection, while^46^ leveraged emoji information to identify the abusive language. Several studies have proposed new techniques and models for improving emoji prediction accuracy.^47^ introduced a label-wise attention mechanism to better understand nuances in emoji prediction tasks, while^48^ incorporated emoji information for tweet classification. Some studies aimed to achieve zero-shot learning for the task using a masked language model. Zhang et al.^49^ explores a multi-task learning approach that focuses on both common and unique aspects for multi-modal sarcasm detection and sentiment analysis in conversational data. By considering shared and distinct features, the proposed model aims to enhance the performance of both tasks in the context of diverse modalities. Zhang et al.^50^ presenting a multitask learning model, this paper addresses the challenges of multimodal sarcasm, sentiment, and emotion recognition in conversational data.

**Table 2:** Succinct overview of the relevant studies categorized by their respective topics.

Topic	Study	Summary
	Eisner et al.^14^	Introduced pre-trained emoji2vec embeddings that analyze emoji usage and relationships between different emojis.
Emoji Prediction	Felbo et al.^15^	Developed models to predict emojis in a text, advancing the understanding of emoji usage in natural language.
	Zhou et al.^16^	Created models for single-label emoji prediction, improving the accuracy of emoji prediction tasks.
	Barbieri et al.^17^	Utilized pre-trained emoji2vec embeddings to create models for single-label emoji prediction, enhancing emoji prediction capabilities.
	Wang et al.^3^	Developed models for multi-lingual emoji prediction using pre-trained emoji2vec embeddings, expanding the scope of emoji prediction to multiple languages.
Sentiment analysis	Al-Baaj et al.^22^	Explored the use of emojis in sentiment analysis, highlighting the role of emojis in conveying sentiment in textual data.
	Chen et al.^23^	Investigated the relationship between emojis and sentiment in social media data, providing insights into sentiment analysis techniques.
	Mao et al.^36^	Introduced a novel gating mechanism for aspect-based sentiment analysis and sequential metaphor identification.
	Santhanam et al.^20^	Utilized emojis to understand crisis events, demonstrating the applicability of emojis in sentiment analysis in emergency situations.
	Mohbey et al.^27^	Employed a hybrid CNN-LSTM approach for sentiment analysis in Monkeypox tweets, improving the accuracy of emotion detection in social media data.
Emotion detection	Zhang et al.^29^	Proposed M3GAT, a multi-modal, multi-task interactive graph attention network, addressing the interdependence of sentiment analysis and emotion recognition in NLP, offering a unified framework for analyzing sentiment and emotion in textual data.
	Ilyas et al.^51^	Focused on emotion detection in code-mixed Roman Urdu and English text, highlighting the challenges and opportunities in detecting emotions in multilingual data.
	Zhang et al.^49^	Explored a multi-task learning approach for multi-modal sarcasm detection and sentiment analysis, demonstrating the effectiveness of incorporating multiple modalities in sentiment analysis tasks.
Multimodal analysis	Xiao et al.^32^	Developed a network for multimodal aspect-based sentiment analysis, integrating textual and visual information to improve sentiment analysis accuracy.
	Zhang et al.^50^	Presented a multitask learning model for multimodal sarcasm, sentiment, and emotion recognition.
	Ilyas et al.^51^	Utilized few-shot learning for emotion detection in code-mixed texts.
	Zhang et al.^52^	Investigated unsupervised learning in NLP tasks, showing promising results in zero-shot settings for various tasks.
Zero-Shot Learning	Tesfagergish et al.^53^	Proposed a two-stage emotion detection method using unsupervised zero-shot learning, demonstrating the potential of zero-shot learning in emotion detection tasks.

Emotion information has also been utilized to improve emoji prediction accuracy, as demonstrated in^54^. Prior investigations, such as the work by Ilyas et al.^51^, have focused on emotion detection in code-mixed Roman Urdu and English text. By leveraging deep learning techniques tailored for code-mixed data, this research contributes to the broader field of sentiment analysis by addressing the unique linguistic challenges posed by code-mixing in emotion detection. In^55^, emojis were used in a framework for identifying offensive language. Furthermore, it was^56^ showed that Support Vector Machine (SVM) models outperform recurrent neural network (RNN) frameworks in emoji prediction tasks. Other studies have explored various ML techniques for emoji prediction and identification. For example,^57^ Gradient Boosting Regression Tree Method (GBM) and bi-directional LSTM for emoji prediction, while^58^ investigated Naive Bayes and RNNs for emoji identification.^59^ leveraged emotion knowledge in emojis to identify affect in tweets. Additionally, some studies proposed novel frameworks, such as a residual CNN-LSTM with attention framework for English emoji prediction in^4^, and a vector similarity-based approach for accurate emoji prediction in^60^. Finally,^61^ introduced a multilingual dataset for sentiment analysis and emoji prediction in Hindi, Bengali, and Telugu, emphasizing the need to explore emoji usage and prediction in different languages and cultures. Overall, these studies highlight the significance and potential of using advanced ML techniques and multimodal information to comprehend and predict emoji usage across various contexts.

Recently, unsupervised learning has gained popularity in the NLP community, showing promising results in zero-shot settings for various tasks^52,62–64^. In emotion recognition, few-shot learning has been used to transfer knowledge from existing datasets to unseen data^65^. To address sentiment analysis,^53^ proposed a two-stage emotion detection method, using an unsupervised zero-shot learning model in the first stage to calculate probabilities for 34 emotions. These probabilities are then used to train a supervised machine learning classifier in the second stage, using ensemble learning to predict sentiment labels. The value of visual, textual, and multimodal emoji prediction has been demonstrated on the MSCOCO dataset^66^ by leveraging the semantic universality of emoji. In addition, to overcome the limitations of small datasets in visual sentiment analysis, the authors in^67^ suggested learning a concise image embedding from abundant data found in social media.

Table 2 provides a succinct overview of the relevant studies categorized by their respective topics in the related work section. In this study, we aim to differentiate ourselves from previous research by developing a multi-task framework that combines sentiment and emotion information to detect multi-label emojis, classify emotions, and analyze sentiment in code-mixed texts. This is the first attempt, to the best of our knowledge, to solve all three tasks simultaneously in a multi-task framework for code-mixed text. We believe that our approach will provide more accurate and comprehensive results compared to previous studies that focus on individual tasks.

## Methodology

In this section, we discuss the proposed *Code-Mixed RMS Fourier Transformer (CM-RFT)* framework for predicting multi-label emojis, sentiment, and emotion on code-mixed inputs.

In the context of English-Hindi code-mixed sentences with labeled examples of emojis and sentiments, the primary objective is to devise a comprehensive multitask framework that should predict multiple emojis in the sentences while simultaneously identifying the corresponding emotion and sentiment expressed by the user. Notably, the emotion detection task is approached in a zero-shot manner, leveraging information acquired from other tasks without direct training.

Let *x* represent an input sentence, $y_e$ signify the associated emotion label, $y_s$ denote the corresponding sentiment label, and $y_m$ encapsulate the set of multiple emojis in the sentence. The multitask framework’s objective functions are defined as follows:

1 $$\begin{aligned} \min _{\theta } \frac{1}{N} \sum _{i=1}^{N} L_m(f_m(x_i; \theta ), y_{m,i}) + \lambda _e L_e(f_e(x_i; \theta ), y_{e,i}) + \lambda _s L_s(f_s(x_i; \theta ), y_{s,i}) \end{aligned}$$

Here, $f_m$, $f_e$, and $f_s$ represent the prediction functions for the emoji, emotion, and sentiment tasks, respectively. The symbol $\theta$ denotes the model parameters, while $\lambda _e$ and $\lambda _s$ serve as regularization coefficients, ensuring a balanced contribution of the emotion and sentiment tasks in the overall objective function. Lastly, $L_m$, $L_s$, and $L_e$ are the respective loss functions for the emoji, sentiment, and emotion tasks.

To illustrate the context, consider the example in Table 3 where we have a code-mixed sentence expressing negative sentiment towards the current state of politics. The emotion conveyed by the user is one of disappointment. The multitask framework aims to predict the corresponding emojis, sentiment, and emotion in such sentences.

The emotion detection task is addressed in a zero-shot manner, meaning it does not undergo direct training but rather relies on information learned from other tasks. In this case, the sentiment of the sentence, which is negative, can inform the emotion detection task about the likely emotion expressed by the user.

**Table 3:** Illustration of a sample data point and the expected output from the proposed framework.

Hinglish input	Predictions
Aaj kal ki raajneeti bahut kaminee ho gayi hai. Feeling disappointed	
with the current state of affairs	Emotion Label: Sadness
	Sentiment Label: Negative
*English Translation:* These days, politics has become very dishonest.	Predicted Emojis:
Feeling disappointed with the current state of affairs.	

### Ethics declarations

We created our resource using publicly available datasets. We adhered to the data use guidelines and did not infringe on any copyright problems. No human participants were involved in our study. Our Institutional Review Board also reviewed and approved this research. We will make the code and data accessible for research purposes through a suitable data agreement mechanism.

## Proposed framework

**Figure 1:**
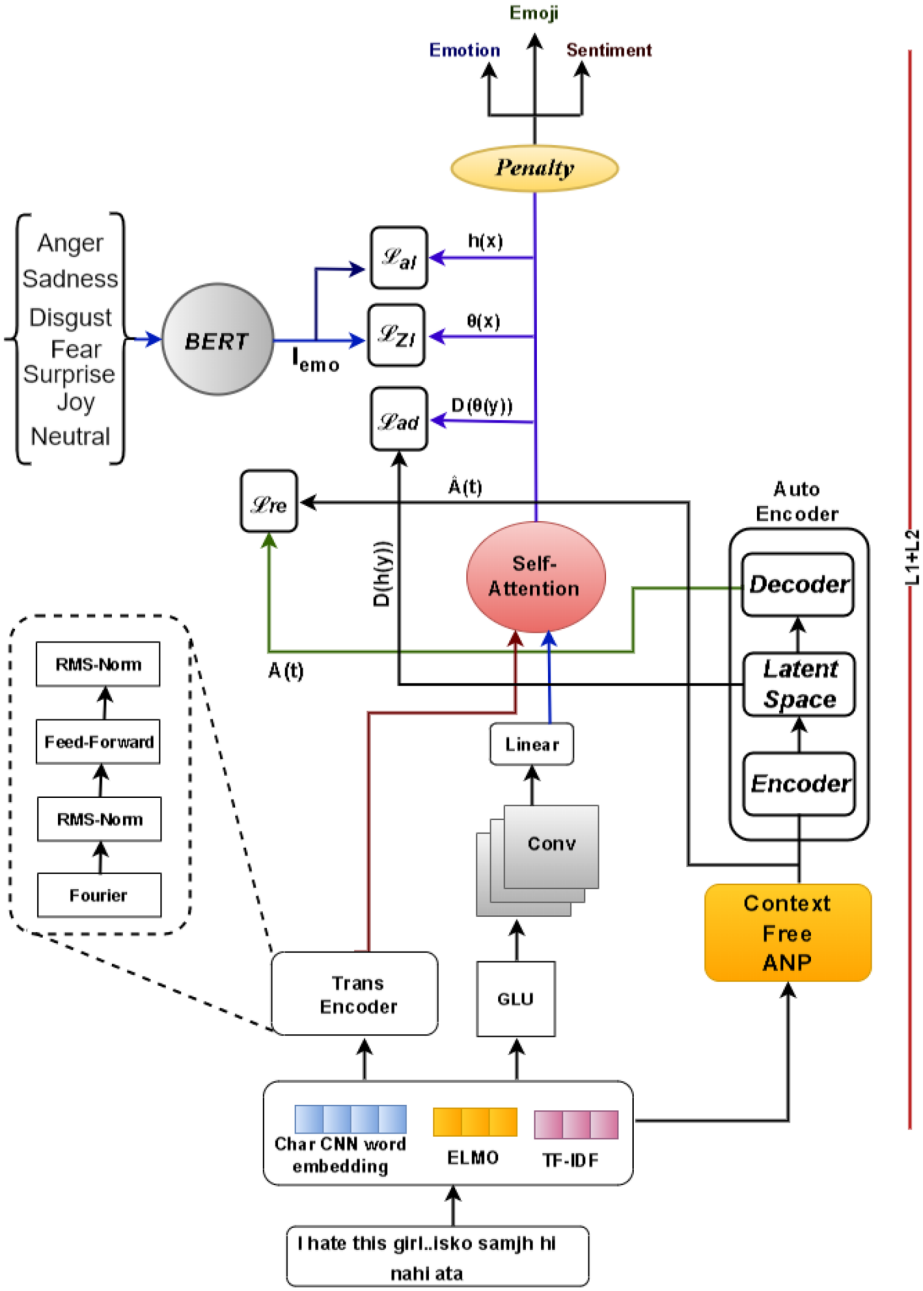
Architectural diagram of our proposed *Code-mixed RMS Fourier Transformer (CM-RFT)* framework.

This section describes the different components involved in the proposed approach for code-mixed textual sentiment analysis. Figure 1 illustrates the overall architecture of our approach.

### SentencePiece tokenizer

In this work, we use SentencePiece^68^ to tokenize tweets. This tool considers tweets as a sequence of Unicode letters and employs byte-pair encoding (BPE)^69^ and unigram language model^70^ to transform sentences into sub-words. The BPE technique begins by including all characters in the dataset in the vocabulary and gradually acquiring a set of merging rules. During the training phase, the unigram language model generates several subword segmentations by selecting them in a probabilistic manner. The output is a set of sub-word sequences, which reduces the dimensionality of the input, especially for rare or unseen words.

### Codemixed embedding generation

Pre-trained word embeddings may not perform well on code-mixed data due to out-of-vocabulary (OOV) words^71^. To address this, we trained word embeddings on the available code-mixed corpus itself. However, choosing the most appropriate embedding model for code-mixed data can be challenging. Therefore, we experimented with three different embedding models and concatenated their outputs for better performance.

#### Char level word embedding

To extract the character-level features for code-mixed data, we follow the work of^72^ and use character-level word embeddings. Since RNN-based encodings are computationally expensive to train and do not significantly outperform convolutional neural networks (CNNs), we utilize a CNN, followed by a max pooling layer, for simplicity of training^73^.

#### Contextual level word embedding

For contextual representation, ELMO^74^ is used, which represents each token as a vector that is dependent on the whole sentence, allowing words to have multiple meanings depending on the context. The product of TF (Term Frequency) and IDF (Inverse Document Frequency) (TF-IDF^75^) of a word is the product of the word’s frequency in the document multiplied by its uniqueness in the corpus of texts. The TF-IDF document frequency model aids in giving the frequent words in the corpus less weight. This method focuses on the unique phrases in the corpus rather than the repeated words, resulting in a more accurate model.

### Code-mixed RMS fourier transformer (CM-RFT)

In this study, we propose the *Code-Mixed RMS Fourier Transformer (CM-RFT)* model, which incorporates the Fourier Transformer model and gated linear unit (GLU) with convolution layer to add more attention with less overhead^76,77^. In the encoder layers, instead of the self-attention mechanism, the Fourier Transformer^77^ is employed. It uses 1D Fourier transforms to transform the input data’s sequence dimension and hidden dimension. The result is a complex number that can be represented as a real number multiplied by the imaginary unit (I), only the real number of the result is stored, avoiding the need to modify the (nonlinear) feed-forward sub-layers or output layers to accommodate complex values.

The encoded output sequence undergoes a residual connection, followed by dropout and normalization, after which it is passed through a position-wise feedforward layer, followed by dropout, a residual connection, and RMS-layer normalization^78^. The RMS-layer norm is a simplified version of LayerNorm that omits the mean-centering process and standardizes layer activations using the root mean square (RMS) statistic, as demonstrated in Eq. (1).

2 $$\begin{aligned} RMS(a) = \frac{1}{n}{\sqrt{\sum _{i=1}^{n}}a_i^2} \end{aligned}$$

The position-wise feedforward layer converts the data from the hidden dimension to $pf_d$, which is often much larger than $h_d$. Following this, a ReLU activation function and dropout are used before returning to a $h_d$ representation. This technique is based on infinitely large neural networks, which improve the ability to approximate and accelerate model optimization.

Applying convolution to the sequence is a logical notion to add extra attention with less overhead^76^. We substitute standard convolution with a lighter variant^79^ composed of linear layers and depth-wise convolution to further minimize computation.

#### Self attention

Next, we apply self-attention (SA) between the Trans-encoder output and the GLU output allowing the model to effectively combine the features learned from both sources of information. The idea behind this is that it could enhance the model’s capacity to capture intricate relationships and patterns within the input data.

#### Auto-encoder

In order to better understand and analyze the sentiment in text data, we utilize a technique called Context-Free-Grammar-Noun-Adjective-Pairs (Context Free ANP). This approach extracts pairs of adjectives and nouns from sentences to capture the meaning of the text. We use a pre-existing implementation of this technique available on GitHub (https://github.com/StatguyUser/Context_Free_Grammar-Noun_Adjective_Pairs) to extract ANP pairs from the text data. In order to learn a latent representation of the ANP pairs, we employ an auto-encoder that takes the ANP features as input and learns a compressed representation of them in the latent space. The proposed method leverages both textual and class semantic knowledge to generate a more precise representation of the emotions conveyed in the text.

To train the auto-encoder, two different loss functions are utilized. The first loss function is the Alignment loss, which aims to minimize the difference between the extracted ANP features and the predicted ANP features. The second loss function is Adversarial loss, which reduces the gap between the encoded class features and the corresponding class centroid in the latent space. The Alignment loss helps to ensure that the ANP pairs extracted from the text are accurately represented in the latent space. The Adversarial loss, on the other hand, encourages the encoded class features to be closer to the corresponding class centroid, which helps to improve the accuracy of the sentiment analysis. The mathematical representation of the Alignment loss and the Adversarial loss is discussed in more detail in the “Training and inference” section of the paper.

#### Penalty

We incorporate a penalty value to improve the prediction of the tokens. The aim is to enhance the model’s understanding of the connection between different labels and the input post. The addition of a penalty in the loss function is motivated by the challenge of defining a decision boundary for token markers in information extraction tasks. This ambiguity makes it difficult for a basic softmax/sigmoid classifier to accurately differentiate between classes and may lead to the misclassification of some samples. The equations below originally represent softmax and sigmoid:

3 $$\begin{aligned} {\mathcal {L}}_{softmax}= -\frac{1}{b_s}\sum _{i=1}^{b_s}\log \frac{\exp ^{{\mathcal {W}} l_i + b_i}}{\sum _{j=1}^N \exp ^{{\mathcal {W}} l_j + b_j}} \end{aligned}$$

4 $$\begin{aligned} {\mathcal {L}}_{sigmoid}= -\frac{1}{b_s}\sum _{i=1}^{b_s} \frac{1}{ \exp ^{{\mathcal {W}} l_i + b_i}} \end{aligned}$$

where $l_i \in {\mathbb {R}}^{d}$ represents the feature of the $i^{th}$ sample, $b_s$ represents the batch size, $b_i$ and $b_j$ represents the bias, and ${\mathcal {W}} \in {\mathbb {R}}^{d*n}$ represents the weight matrix.

To address the difficulty in determining the decision boundary for token markers in information extraction tasks, the Insightface loss technique^80^ can be used to normalize the feature $l_i$ and weight matrices ${\mathcal {W}}$, and assess feature similarity based on the angle difference between them. The loss function is updated by adding a penalty value *x* to the angle $\theta$, which helps to converge the feature more quickly. The loss function is updated for both softmax and sigmoid as follows:

5 $$\begin{aligned} {\displaystyle {\mathcal {L}}_{softmax}}= {\displaystyle -\frac{1}{b_s}\sum _{i=1}^{b_s}\log } \frac{\exp ^{a(cos(\theta +x))}}{\displaystyle {\exp ^{{a(cos(\theta +x))}}+\sum _{j=1}^N \exp ^{{a(cos(\theta ))}}}} \end{aligned}$$

6 $$\begin{aligned} {\mathcal {L}}_{sigmoid}= -\frac{1}{b_s}\sum _{i=1}^{b_s} \frac{1}{\exp ^{{a(cos(\theta +x))}}+\exp ^{{a(cos(\theta ))}}} \end{aligned}$$

In the given context, $\theta$ indicates the angle between the weight ${\mathcal {W}}$ and the feature $l_i$, and *a* is the amplifier function. The equation $\exp ^{{a(cos(\theta +x))}}$ is used to calculate the similarity score of the positive sample, whereas $\exp ^{{a(cos(\theta ))}}$ is used to calculate the similarity score of the negative samples. The penalty value *x* adds a margin to the classification boundary to improve the convergence rate of the feature.

## Training and inference

In this section, we outline the process of training our model and explain how to make predictions for emotions. Our model is trained in an end-to-end fashion using four different loss functions.

### Reconstruction loss

Our goal is to align the structures of label features and adjective-noun pair features in the learned latent space through the use of an auto-encoder. The auto-encoder will reconstruct adjective-noun pair features and generate latent features while preserving the emotion-related information. The optimization of the auto-encoder parameters is performed by reducing the loss function, which measures the similarity between the input and output of the auto-encoder.

$$\begin{aligned} {\mathcal {L}}_{re}={||{\hat{A}}((t))-A((t))||}^2_2 \end{aligned}$$

The input and output embedding features of the auto-encoder are represented by ${\hat{A}}$ and *A* respectively.

### Alignment loss

We aim to align the latent space and label semantic spaces of an auto-encoder so that the label representations produced are more related to latent emotion concepts. This is achieved by optimizing the following loss function:

$$\begin{aligned} {\mathcal {L}}_{al}={||h(x)-\phi {(l_{emo})||}^2_2} \end{aligned}$$

where h(x) defines the latent space embedding generated by the auto-encoder, and $l_{emo}$ defines the emotion embedding. The overall objective function is realized by combining alignment loss and reconstruction loss.:

$$\begin{aligned} {\mathcal {L}}_{re}+{\mathcal {L}}_{al} \end{aligned}$$

### Emotion features

We employ the pre-trained BERT (*base*)^81^ model to encode the semantic feature information for the Ekman’s^82^ basic emotion classes (*Anger*, *Disgust*, *Sad*, *Joy*, *Surprise*, *Fear*, *Fear*). Additionally, we consider the *Neutral* class to accommodate instances that do not fall in the scope of Ekman’s categorization. Fetching the features from BERT obviates the need for further human annotation.

### Zero-shot loss

The objective of our model is to minimize the difference between the combined feature of text represented by $\theta (x)$, and the semantic feature of the label, represented by $\phi {(l_{emo})}$, through optimization.

$$\begin{aligned} {\mathcal {L}}_{zl}={||\theta (SA(x)-\phi {(l_{emo})}||}^2_2 \end{aligned}$$

### Adversarial loss

We aim to minimize the distance between the discriminative capacity of the text ($\theta (x)$ which represents *SA*(*t*)) and the rich emotional structural data contained in the feature $\phi (l_{emo})$. This is achieved through the use of an adversarial restriction that aims to fool the discriminator network ${\mathcal {D}}$ so that the output features of $A(\theta (x))$ are as indistinguishable from the ANP features as possible.

$$\begin{aligned} {\mathcal {L}}_{ad}={\mathcal {E}}_y(\log {\mathcal {D}}(h(y))-{\mathcal {E}}_y(\log {\mathcal {D}}(\theta (y)) \end{aligned}$$

where $\theta (y)$ defines the feature of text, and *h*(*y*) defines the latent feature space.

### Joint Loss

Our model is trained using a combination of the various loss functions described earlier.

$$\begin{aligned} {\mathcal {L}}_{joint}= {\mathcal {L}}_{ad}+{\mathcal {L}}_{zl}+({\mathcal {L}}_{re}+{\mathcal {L}}_{al}) \end{aligned}$$

### Label prediction

When presented with a test set of tweet, which consist of an text, and a collection of label that include emotion, our model will perform label classification through a straightforward nearest neighbour (NN) search. The test tweets and labels are input into the embeddings to produce $\theta (t)$ and $\phi {(l_{emo})}$.

$$\begin{aligned} {||\theta (t)-\phi {(l_{emo})}||}^2_2 \end{aligned}$$

### Model training

We train our model through an unified loss function as shown in Eq. (7).

7 $$\begin{aligned} L_{total}=L_{m}+L_{e}+L_{s} \end{aligned}$$

$L_m$ is the loss function for the emoji task, which can be defined as the sum of binary cross-entropy loss for each predicted emoji:

8 $$\begin{aligned} L_m = -\sum _{j=1}^{k} y_{m,i,j} \log ({\hat{y}}_{m,i,j}) + (1 - y_{m,i,j}) \log (1 - {\hat{y}}_{m,i,j}) \end{aligned}$$

where *k* is the number of possible emojis, $y_{m,i,j}$ is the binary label for the *j*-th emoji in the *i*-th sentence, and ${\hat{y}}_{m,i,j}$ is the predicted probability of the *j*-th emoji.

$L_e$ is the loss function for the emotion task, which can be defined as the negative log-likelihood of the predicted emotion:

9 $$\begin{aligned} L_e = -\log \frac{\exp (f_e(x_i; \theta ){y_{e,i}})}{\sum _{j=1}^{c} \exp (f_e(x_i; \theta )_j)} \end{aligned}$$

where *c* is the number of possible emotions and $f_e(x_i; \theta )_j$ is the predicted score for the *j*-th emotion.

$L_s$ is the loss function for the sentiment task, which can be defined as the binary cross-entropy loss:

10 $$\begin{aligned} L_s = -y_{s,i} \log ({\hat{y}}_s) - (1 - y_{s,i}) \log (1 - {\hat{y}}_s) \end{aligned}$$

where $y_{s,i}$ is the binary label for the sentiment of the *i*-th sentence, and ${\hat{y}}_s$ is the predicted probability of positive sentiment.

## About dataset

This research builds upon the SentiMix dataset (available at https://zenodo.org/record/3974927#.ZEva4pFBxH6), initially introduced in Task 9 of the SemEval 2020 shared task^12^. Comprising approximately 20,000 tweets, this dataset is partitioned into train, test, and development sets, each annotated with one sentiment label (positive, negative, or neutral).

**Figure 2:**
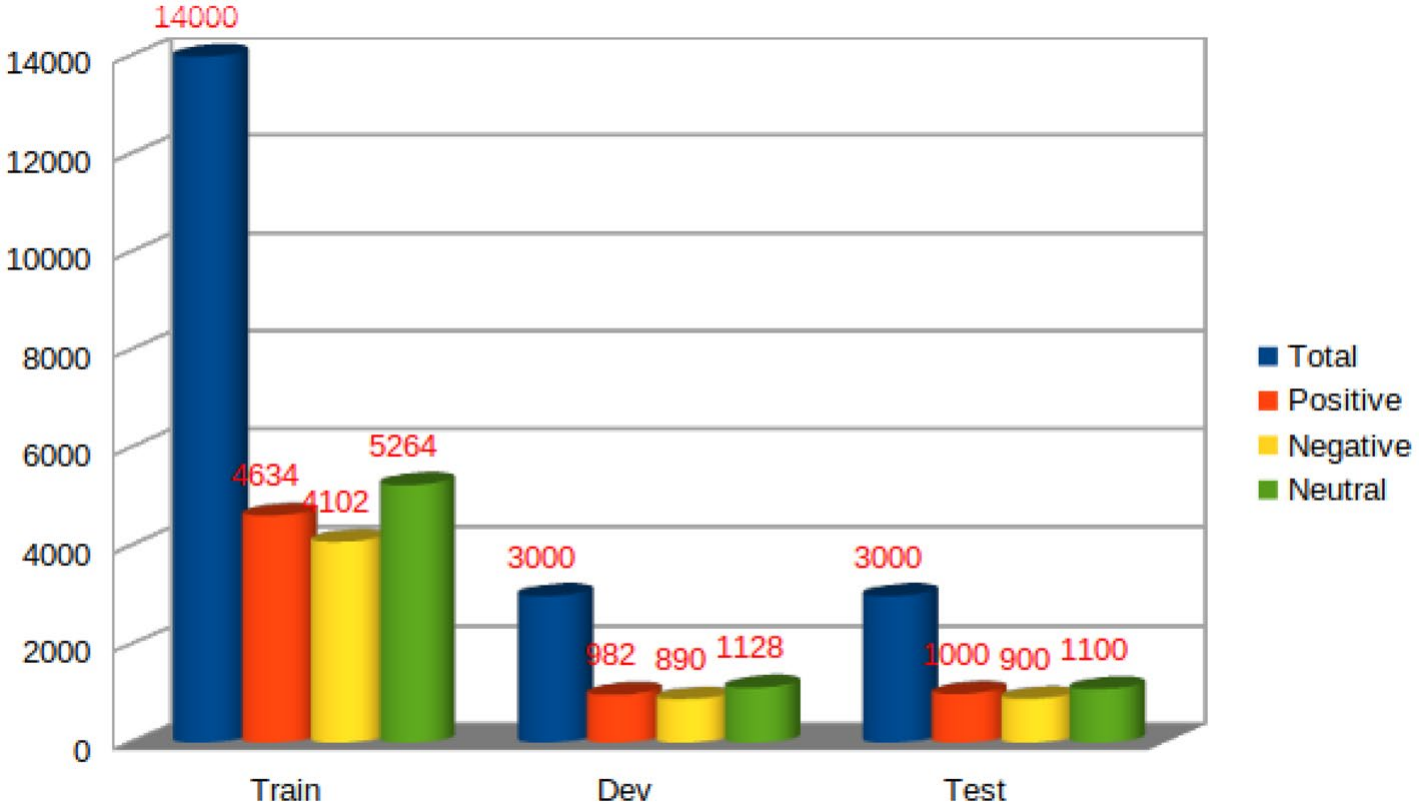
Distribution of instances in the *SENTIMOJI* dataset over the sentiment classes.

To facilitate investigations into the interconnectedness of multiple emojis, sentiment, and emotion within code-mixed text, we introduce the *SENTIment and eMOJI (SENTIMOJI)* dataset. The sentiment task’s data distribution across the train, test, and development sets is depicted in Fig 2. Furthermore, detailed statistics regarding the dataset can be found in Table 4.

The key highlights of the SENTIMOJI dataset are:

- The *SENTIMOJI* dataset is meticulously designed to accommodate the complexities of code-mixed language.
- It includes multi-label annotations for emojis, offering a richer understanding of sentiment expression.
- Annotations encompass sentiment and emotion information, providing a holistic view of user expression.
- The dataset spans across various platforms, ensuring diversity in the data sources.

### Data annotation

#### Emoji annotation

The process of annotating multiple emojis for each text involved three annotators. Each annotator was tasked with labeling a given text with emojis that best represented the sentiment expressed. To ensure consistency, annotators were provided with a predefined set of emojis, specifically the top 64 emojis from the emoji set mentioned in^83^. These emojis served as the pool from which annotators could select while labeling the text.

The annotation process was conducted independently, with annotators prohibited from communicating with each other to avoid bias or influence. Each instance from the SentiMix dataset, including samples from the train, test, and validation sets, was presented to the annotators for labeling. Their annotations were then compared to assess the level of agreement. In cases where discrepancies arose, a discussion among annotators or consultation with a senior annotator was utilized to resolve disagreements.

The final labels were determined based on the consensus reached among the annotators. Overall, the annotation process achieved an impressive Fleiss-Kappa score of 0.75, indicating a high level of reliability. Notably, the selected emojis (, , , , , , , , , , , ,, ) used for labeling the SentiMix dataset encompassed a diverse range of emotions and sentiments.

**Table 4:** Dataset statistics of our Mu-Emoji dataset.

Statistics	Train	Valid	Test
# CMI(codemixed index)	25.32	25.53	25.13
# of Utterances	14,000	3,000	3,000
# of Positive class	4,634	982	1,000
# of Neutral class	5264	1,128	1,100
# of Negative class	4102	890	900
Average # of emojis per utterance	4	3	3
# of unique tokens	7781	1189	2458

**Table 5:** Test Sample instances from the *SENTIMOJI* Dataset.

Utterance	English Translation	Emoji 1	Emoji 2	Emotion	Sentiment
@ Reema sahanii suar tere jaiso ko sikhaya ja hi nahi	@ Reema sahanii Pig people like you never learn anything				
sakta because u r suar hai	because you are Pig			Disgust	Negative
@ Payal _ Rohatgi @ YouTube Tweets people do when	@ Payal _ Rohatgi @ YouTube Tweets people do when				
they are irrelevant to the point ki koi ghaas nahi dalta	they are irrelevant to the point no one gives u the shit			Anger	Negative
@ redribbonmusik Zindagi har lamha hai khushi kar	@ redribbonmusik *random song* I love this				
isse dosti o yaara ve I love this yaara				Joy	Positive
meta Thank you so much apka ek new india banane ke liye Koti koti pradam apko	meta Thank you so much for creating a new India			Joy	Positive

#### Emotion annotations

In addressing the emotion detection task, we adopted a zero-shot approach, enabling model training without specifically labeled emotion data. However, to evaluate the efficacy of our model, we manually annotated each instance in the SentiMix test set with an emotion class based on Ekman’s six basic emotions: anger, disgust, fear, joy, sadness, and surprise. Additionally, we included a neutral class to accommodate instances that did not neatly fit within Ekman’s classification scheme.

The annotation process for emotion detection was carried out independently by annotators, and inter-annotator agreement was measured using the Fleiss-Kappa score, yielding a commendable score of 0.78, indicative of high-quality annotations. Sample annotated instances from the SENTIMOJI test set, illustrating the assigned emotion classes, are presented in Table 5.

## Dataset validation

The validity of the SENTIMOJI dataset is ensured through a meticulous validation process utilizing an Emoji matrix. This matrix cross-references annotations to mitigate any discrepancies, thereby enhancing the precision and reliability of the dataset for subsequent analysis and model training. The Emoji matrix is a matrix that defines the semantics of different emojis, inspired by the lexical matrix of WordNet. It consists of 14 columns, each representing a different emoji considered for the task, and rows representing the different keywords associated with that emoji. Table 6 shows the different emojis and their corresponding keywords.

Each cell in the matrix had a value of either 1 or 0, depending on whether the description of the emoji contained the associated keyword or not. More specifically, if the description of the emoji contained a particular keyword, the corresponding cell in the matrix was assigned a value of 1, and if not, it was assigned a value of 0.

The emoji matrix is created in such a way that its rows represent the set of keywords (k_1, k_2, ..., k_n), and columns represent different emojis (e_1, e_2, ..., e_m), as illustrated in Table 6. The value of each cell in the matrix is determined by:

11 $$\begin{aligned} Y_{e,k}= {\left\{ \begin{array}{ll} 1 \text {, if the description of } e_j \text {contains } k_i \ 0, otherwise \end{array}\right. } \end{aligned}$$

This matrix helps in identifying the polysemy and synonymy nature of the emojis and keywords, respectively. Polysemy refers to the idea that an emoji can have multiple keywords to convey its meaning, while synonymy means that a particular keyword may be used to represent multiple emojis.

**Table 6:** Different keys for the emojis *SENTIMOJI* Dataset.

Emojis	Meaning	Keywords
	*Red Face*	*Anger, Hurt, Rage, Madness*
	*Neutral Face*	*Mild irritation and concern or a deadpan sense of humor*
	*Face Blowing a Kiss*	*Kiss, Blowing Kiss, Heart, Love, Lips*
	*Pensive Face*	*Disappointed, Hurt or Lonely*
	*Pouting Face*	*Anger, Hurt, Rage, Madness*
	*Disappointed*	*Disappointed, Grief, Stress, Regret, and Remorse*
	*Sad but Relieved Face*	*Sad, Relieved (things are not that bad)*
	*Vomiting Emoji*	*Physical illness, Disgust*
	*Nauseated Face*	*Physical illness, Disgust*
	*Face with tears*	*Tears, Joy, Happy*
	*Red Heart*	*Heart, Love, Passion, Romance, Intense, Desire, Death, Evil, Cold*
	*Rolling on the Floor*	*Tears, Joy, Happy*

**Table 7:** Comparison with existing datasets.

Dataset/Paper	Codemixed	Sentiment	Emoji	
			Single	Multi-label
Multi-resolution Annotations for Emoji Prediction^84^	✗	✗	✓	✓
DeepMoji^15^	✗	✗	✓	✓
SemEval 2018 Task 2:Multilingual Emoji Prediction^1^	✗	✗	✓	✗
Are Emojis Predictable?^17^	✗	✗	✓	✗
The emojification of sentiment on social media: Collection and analysis of a longitudinal Twitter sentiment dataset^85^	✗	✓	✓	✗
*SENTIMOJI (Ours)*	✓	✓	✓	✓

In order to assess the effectiveness of our proposed framework, we utilized equivalent groups in place of the original emojis, and substituted the predicted emoji by the corresponding group as determined by the Emoji matrix. This constituted our final prediction. By validating the Emoji matrix, we ensured the precision of our annotated dataset, which gave us confidence in using this data for subsequent analysis.

### Comparison with existing datasets

We compare our proposed dataset, *SENTIMOJI*, with existing datasets for emoji prediction. Table 7 summarizes the comparison. The dataset proposed in^84^ and^15^ contains single and multi-label emoji annotations for English tweets, whereas^1^ proposed a single-label emoji dataset for two languages. More recently, in^85^, an emoji and sentiment annotated dataset was proposed for English tweets. Compared to these existing datasets, *SENTIMOJI* is unique because it is code-mixed in nature, containing English-Hindi code-mixed tweets. Moreover, *SENTIMOJI* not only contains multi-label emoji annotations, but it also includes sentiment and emotion information, making it a more comprehensive dataset for emoji prediction. The inclusion of sentiment and emotion information is essential as emojis are often used to convey these aspects of the message.

### Dataset significance

The significance of the *SENTIMOJI* dataset lies in its capacity to overcome the limitations of existing resources. Notably, *SENTIMOJI* stands out as a unique code-mixed dataset, providing multi-label emoji annotations alongside sentiment and emotion information. As illustrated in Table 7, our dataset boasts comprehensive statistics, showcasing its extensive and diverse range of emojis, making it the largest multi-label emoji dataset known to date. By embracing code-mixed language and offering multi-label annotations for emojis alongside sentiment and emotion data, *SENTIMOJI* paves the way for more nuanced analysis and model development. This dataset emerges as an indispensable asset for researchers and practitioners striving to gain comprehensive insights into sentiment expression within code-mixed contexts.

### Experimental setup

Our study focuses on a multi-task framework that addresses three distinct tasks: emoji detection, sentiment detection, and emotion detection. We define the experimental setups for each task as follows.

- Emoji Classification ($${mE}_{C}$$)
  - The *SENTIMOJI* dataset contains twelve different emojis, and each tweet can be associated with more than one emoji.
- Sentiment Classification ($${S}_{C}$$):
  - Each tweet in the dataset is associated with one of three sentiment classes: positive, neutral, or negative.
  - We utilize one-hot vectors to represent the sentiment classes that correspond to each tweet.
- Emotion Classification ($${E}_{C}$$):
  - Each tweet in the dataset is associated with one of five emotion classes: anger, sadness, disgust, fear, surprise, or joy, as well as a neutral class.
  - We utilize one-hot vectors to represent the emotion classes that correspond to each tweet.

## Experiments, results and analysis

Extensive discussion of experiments, results, and analysis on our introduced dataset for the proposed method and existing state-of-the-art baselines are presented below.

### Baselines

The following baseline methods are compared to our proposed approach.

- *XLMR*$^{[FT+LS+RF]}$^86^: In this method, a pre-trained BERT (Bidirectional Encoder Representations from Transformers) model is fine-tuned (FT) to perform sentiment analysis. To reduce overfitting, the authors incorporated label smoothing (LS) and rule-based features (RF) such as negation handling and sentiment shift detection. This model is used for emoji, sentiment, and emotion analysis tasks.
- Multilingual BERT (mBERT)^87^: The authors utilized a transformer-based language model called mBERT to learn contextual embeddings for words in multiple languages. mBERT was pre-trained on large amounts of monolingual and multilingual text data and fine-tuned on the SentiMix code-mixed dataset for sentiment detection and emotion recognition.
- XLMR$^{MTL}$^87^: The authors used XLM-R, a cross-lingual language model based on a transformer architecture that was pre-trained on a larger dataset including code-mixed text. XLM-R can encode and decode text in multiple languages and has achieved state-of-the-art results on various NLP tasks, including sentiment analysis and emotion recognition. They fine-tuned XLM-R on the SentiMix code-mixed dataset for sentiment detection and emotion recognition.
- *TL-XLMR*$^{[LS]}$^6^: To detect sentiment and recognize emotions in the SentiMix code-mixed dataset, the authors employed an end-to-end multitask framework based on a transformer architecture. They fine-tuned XLM-RoBERTa (XLMR), a pre-trained cross-lingual embedding model, with task-specific data to improve model efficiency through transfer learning.
- *TL-mBERT*$^{[LS]}$^6^: In this ablation experiment, the authors replaced the XLMR module with mBERT to investigate the significance of the sentence encoder in *TL-XLMR*$^{[LS]}$. The model was fine-tuned on the SentiMix code-mixed dataset to perform sentiment detection and emotion recognition.

### Implementation details

Our suggested model is put into practice using PyTorch, a well-liked Python deep-learning toolkit. We employ the F1-score (F1) as our evaluation metric for both emotion and sentiment prediction and for emoji we used Jaccord Index (JI), macro F1-score. We utilize Adam optimizer^88^ and do a grid search for 200 epochs to improve the model. We use Transformer Encoder with two layers our embedding size is 300 which we find empirically (checked for 100, 150, 200 and 300). The dropout rate is set at 0.5 while the learning rate is set at 0.05. The auto-latent encoder’s dimension was found to be 2048 using empirical techniques. The discriminator, ${\mathcal {D}}$, is composed of two fully connected layers, a ReLU layer. The learning rate is set to 1e-3, weight decay of 1e-4, and momentum of 0.3. By contrasting the F1 and accuracy scores with different baselines, the efficacy of our strategy is assessed. In the *CM-RFT*, the kernel is dynamically computed from the input using a fully connected layer. The kernel sizes are [3, 5, 7, 31*3], and each module has 4 heads (half the number of heads in the transformer base model).

#### Evaluation metrics

For the emoji detection tasks, we consider the *Jaccard Index (JI)*^89^ and *Hamming loss (HL)*^90^ metrics to evaluate the performance of our proposed system. Additionally, we also report the *micro-averaged F1*^91^ score and Accuracy values for the same (as shown in Table 8). JI, HL, and micro-averaged F1 are popular choices to evaluate multi-label classification tasks. For the sentiment and emotion detection tasks (as shown in Tables 9 and  10), we report the macro-averaged F1 score^91^ and accuracy values for our proposed model.

- *Micro-averaged F1 score:* For multi-label classification tasks, the micro-averaged F1 score is a commonly used metric that computes the F1 score globally by counting the true positives (TP), false negatives (FN), and false positives (FP) across all labels. The formula for the micro-averaged F1 score is: $F1_{micro} = \frac{2 * \sum _{i=1}^n TP_i}{2 * \sum _{i=1}^n TP_i + \sum _{i=1}^n FP_i + \sum _{i=1}^n FN_i}$
- *Macro-averaged F1 score:* The macro-averaged F1 score is another commonly used metric for multi-label classification tasks. It computes the F1 score for each label and then takes the average of these F1 scores. The formula for the macro-averaged F1 score is: $F1_{macro} = \frac{1}{n} \sum _{i=1}^n \frac{2 * TP_i}{2 * TP_i + FP_i + FN_i}$
- *Accuracy:* Accuracy is a metric that measures the proportion of correctly classified labels to the total number of labels. The formula for accuracy is: $A = \frac{\sum _{i=1}^n TP_i}{\sum _{i=1}^n TP_i + \sum _{i=1}^n FP_i}$
- *Hamming Loss:* The Hamming loss measures the proportion of misclassified labels to the total number of labels. The formula for Hamming loss is: $HL = \frac{1}{n} \sum _{i=1}^n \frac{xor(Y_i, \hat{Y_i})}{m}$ where *n* is the number of instances, *m* is the number of labels, $Y_i$ is the true label vector for instance *i*, $\hat{Y_i}$ is the predicted label vector for instance *i*, and *xor* is the logical XOR operator.
- *Jaccard Index:* The Jaccard Index measures the similarity between two sets by computing the ratio of the size of their intersection to the size of their union, and it is used to measure the similarity between the predicted and true label sets in multi-label classification. The formula for the Jaccard Index is: $JI = \frac{1}{n} \sum _{i=1}^n \frac{|Y_i \cap \hat{Y_i}|}{|Y_i \cup \hat{Y_i}|}$ where *n* is the number of instances, $Y_i$ is the true label set for instance *i*, and $\hat{Y_i}$ is the predicted label set for instance *i*. The Jaccard similarity is computed as the size of the intersection of the predicted and true label sets divided by the size of their union. The resulting score ranges from 0 to 1, with 1 representing the perfect similarity between the predicted and true label sets.

### Results

Tables 8,  9, and  10 present the performance of CM-T, CM-FT, and *CM-RFT* models for the emoji, sentiment, and emotion tasks in UTL, DTL, and TTL setups. These setups investigate the effectiveness of multi-task learning in improving overall system performance compared to single-task learning.

**Table 8:** Results of our proposed *CM-RFT* framework for Multi-label Emoji Classification. The maximum scores are displayed in bold. $S_C$: Sentiment classification, $E_C$: Emotion classification, $mE_C$: multi-label Emoji classification, CE: Character embeddings, A: Accuracy, HL: Hamming loss, JI: Jaccard Index.

Tasks		Embeddings			CM-T				CM-FT				CM-RFT			
		CE	Elmo	TF-IDF	F1 (%)	A (%)	HL	JI	F1 (%)	A (%)	HL	JI	F1 (%)	A (%)	HL	JI
UTL	$mE_C$	$\surd$	–	–	0.56	0.58	0.19	0.43	0.57	0.60	0.19	0.45	0.59	0.62	0.15	0.52
	$mE_C$	$\surd$	$\surd$	–	0.57	0.59	0.18	0.44	0.60	0.62	0.18	0.47	0.62	0.65	0.14	0.54
	$mE_C$	$\surd$	$\surd$	$\surd$	0.59	0.62	0.18	0.46	0.63	0.65	0.17	0.49	0.64	0.67	0.13	0.56
DTL	$S_C$+$mE_C$	$\surd$	–	–	0.58	0.61	0.16	0.53	0.60	0.63	0.13	0.55	0.63	0.65	0.11	0.58
	$S_C$+$mE_C$	$\surd$	$\surd$	–	0.61	0.63	0.15	0.52	0.64	0.67	0.12	0.56	0.65	0.69	0.09	0.60
	$S_C$+$mE_C$	$\surd$	$\surd$	$\surd$	0.62	0.64	0.14	0.54	0.65	0.68	0.11	0.58	0.68	0.71	0.07	0.61
	$mE_C$+$E_C$	$\surd$	–	–	0.59	0.60	0.17	0.47	0.60	0.63	0.15	0.50	0.60	0.64	0.13	0.55
	$mE_C$+$E_C$	$\surd$	$\surd$	–	0.62	0.63	0.16	0.49	0.62	0.64	0.14	0.52	0.64	0.66	0.11	0.57
	$mE_C$+$E_C$	$\surd$	$\surd$	$\surd$	0.61	0.64	0.14	0.51	0.64	0.67	0.13	0.54	0.67	0.69	0.09	0.59
TTL	$S^C$+$E_C$+$mE_C$	$\surd$	–	–	0.60	0.65	0.12	0.56	0.63	0.67	0.064	0.62	0.67	0.74	0.059	0.64
	$S^C$+$E_C$+$mE_C$	$\surd$	$\surd$	–	0.61	0.65	0.10	0.59	0.65	0.68	0.061	0.64	0.69	0.73	0.057	0.67
	$S^C$+$E_C$+$mE_C$	$\surd$	$\surd$	$\surd$	0.63	0.66	0.09	0.60	0.67	0.70	0.056	0.66	0.73	0.75	0.054	0.69

#### Multi-label emoji classification ($mE_C$):

The results reported in Table 8 are the performance metrics of three different models (CM-T, CM-FT, *CM-RFT*) trained on three different setups (uni-task learning, dual-task learning, and tri-task learning) for the task of emoji detection.

In the uni-task learning setup, where each task is solved individually, the performance of the *CM-RFT* model improves as more features are added. Specifically, the performance improves as we go from using only character embeddings to character embeddings + Elmo embeddings + TF-IDF. The F1 score increases from 0.59 to 0.64, the accuracy score from 0.62 to 0.67, while the hamming loss decrease from 0.15 to 0.13, and the Jaccard index increases from 0.52 to 0.56. These results suggest that using multiple features can improve the performance of the emoji detection task.

In the dual-task learning setup, where the emoji task is jointly learned with sentiment/emotion tasks are jointly learned, the performance of the *CM-RFT* model further improves compared to the uni-task learning setup. The improvement is more evident when the model is trained on Character embeddings + Elmo embeddings + TF-IDF features. The F1 score increases from 0.64 to 0.68, the accuracy score from 0.67 to 0.71, while the Hamming loss decrease from 0.13 to 0.07, and the Jaccard index increases from 0.56 to 0.61, respectively. These results suggest that training the model on multiple tasks can lead to further improvements in the performance of the emoji detection task.

In the tri-task learning setup, where sentiment, emotion, and emoji detection tasks are jointly learned, the performance of the *CM-RFT* model improves even further compared to the dual-task learning setup. The F1 score increases from 0.68 to 0.73, the accuracy score from 0.71 to 0.75, while the Hamming loss decrease from 0.07 to 0.054, and the Jaccard index increases from 0.61 to 0.69. These results suggest that joint learning of multiple tasks leads to significant improvements in the performance of the emoji detection task.

Overall, the results suggest that the performance of the emoji detection task can be improved by using multiple features and by training the model on multiple tasks. Additionally, the results suggest that sentiment and emotion have a significant impact on the performance of the emoji detection task as joint learning of these tasks leads to significant improvements in performance.

**Table 9:** Results of our proposed *CM-RFT* framework for sentiment classification. State-of-the-art result was 0.75 for sentiment, described in^12^. The maximum scores are displayed in bold. $S_C$: Sentiment classification, $E_C$: Emotion classification, $mE_C$: multi-label Emoji classification, CE: Character embeddings, A: Accuracy, HL: Hamming loss, JI: Jaccard Index.

Tasks		Embeddings			CM-T		CM-FT		CM-RFT	
		CE	Elmo	TF-IDF	F1 (%)	A (%)	F1 (%)	A (%)	F1 (%)	A (%)
UTL	$S_C$	$\surd$	–	–	64.21	64.93	65.89	67.11	69.49	71.55
	$S_C$	$\surd$	$\surd$	–	66.69	68.17	68.32	68.79	71.88	73.34
	$S_C$	$\surd$	$\surd$	$\surd$	68.24	69.34	70.11	71.78	72.65	75.19
	$S_C$+$mE_C$	$\surd$	–	–	67.26	71.82	69.43	72.91	74.69	76.71
DTL	$S_C$+$mE_C$	$\surd$	$\surd$	–	70.31	71.81	71.72	73.48	76.41	77.35
	$S_C$+$mE_C$	$\surd$	$\surd$	$\surd$	71.42	74.34	73.34	75.51	78.22	79.21
	$S_C$+$E_C$	$\surd$	–	–	66.13	68.79	68.81	70.39	72.21	74.33
	$S_C$+$E_C$	$\surd$	$\surd$	–	67.71	70.19	69.29	71.32	73.98	75.87
	$S_C$+$E_C$	$\surd$	$\surd$	$\surd$	69.83	71.98	71.31	73.10	74.64	77.31
TTL	$S_C$+$E_C$+$mE_C$	$\surd$	–	–	71.98	72.79	73.31	74.53	78.54	79.27
	$S_C$+$E_C$+$mE_C$	$\surd$	$\surd$	–	72.51	75.35	74.89	77.49	81.11	81.93
	$S_C$+$E_C$+$mE_C$	$\surd$	$\surd$	$\surd$	73.31	78.43	75.42	79.26	**82.35**	**83.14**

#### Sentiment classification ($S_C$)

The sentiment classification task results are presented in Table 9 for the joint learning of emotion and emoji tasks. In the uni-task setup, where each task is performed independently, the *CM-RFT* model achieves the highest performance for the sentiment task with an F1 score of 72.65 and accuracy of 75.19. This suggests that including extra features, such as Elmo embeddings and TF-IDF features, can enhance sentiment detection performance across all models compared to those utilizing only character embedding features.

In the dual-task setup, when sentiment and emoji tasks are jointly learned, the F1 score and accuracy score of the sentiment detection task improve from 72.65 and 75.19, respectively, in the uni-task setup to 78.22 and 79.21, respectively, when using character embeddings, Elmo embeddings, and TF-IDF features. Similarly, when sentiment and emotion tasks are jointly learned, the F1 score and accuracy score of the sentiment detection task improve from 72.65 and 75.19, respectively, in the uni-task setup to 74.64 and 77.31, respectively, when using character embeddings, Elmo embeddings, and TF-IDF features.

In the tri-task setup, where sentiment, emotion, and emoji detection tasks are solved jointly, the *CM-RFT* model achieves the best performance for the sentiment task with an F1 score of 82.35 and accuracy of 83.14, followed by the CM-FT model with an F1 score of 75.42 and accuracy of 79.26. This again confirms that multitask learning helps to improve sentiment detection performance when it is learned jointly with other tasks.

The findings indicate that integrating emotion and emoji detection tasks into the sentiment classification task can enhance the model’s performance. The tri-task learning setup demonstrated the highest performance for the sentiment task, implying that incorporating these extra tasks can improve the model’s comprehension of the sentiment expressed in text. The enhanced performance is likely due to the additional contextual information that emotions and emojis provide, particularly in cases where the sentiment is complicated or sarcastic. Therefore, incorporating emotion and emoji detection tasks could be a useful technique for enhancing the performance of sentiment classification models. Moreover, incorporating additional features, such as Elmo embeddings and TF-IDF features, can also improve the sentiment detection performance.

**Table 10:** Results of our proposed *CM-RFT* framework for Emotion Classification. The maximum scores are displayed in bold. $S_C$: Sentiment classification, $E_C$: Emotion classification, $mE_C$: multi-label Emoji classification, CE: Character embeddings, A: Accuracy.

Tasks		Embeddings			CM-T		CM-FT		CM-RFT	
		CE	Elmo	TF-IDF	F1 (%)	A (%)	F1 (%)	A (%)	F1 (%)	A (%)
UTL	$E_C$	$\surd$	–	–	46.21	48.54	47.54	49.22	48.78	52.77
	$E_C$	$\surd$	$\surd$	–	48.53	50.11	50.86	52.67	52.31	54.54
	$E_C$	$\surd$	$\surd$	$\surd$	50.32	52.79	52.67	54.72	54.21	56.79
	$S_C$+$E_C$	$\surd$	–	–	52.84	53.26	52.91	54.19	54.82	57.73
DTL	$S_C$+$E_C$	$\surd$	$\surd$	–	52.98	55.03	54.41	56.91	55.41	58.91
	$S_C$+$E_C$	$\surd$	$\surd$	$\surd$	53.92	56.31	55.23	58.92	57.33	61.22
	$mE_C$+$E_C$	$\surd$	–	–	48.42	51.12	50.32	52.81	52.83	55.95
	$mE_C$+$E_C$	$\surd$	$\surd$	–	50.42	53.80	52.72	54.30	53.25	56.15
	$mE_C$+$E_C$	$\surd$	$\surd$	$\surd$	51.21	54.79	54.11	56.90	55.76	58.63
TTL	$S_C$+$E_C$+$mE_C$	$\surd$	–	–	53.88	57.26	56.19	58.37	57.87	59.54
	$S_C$+$E_C$+$mE_C$	$\surd$	$\surd$	–	55.21	57.82	57.51	59.71	59.33	61.91
	$S_C$+$E_C$+$mE_C$	$\surd$	$\surd$	$\surd$	56.79	59.18	58.71	61.63	**60.53**	**63.73**

#### Emotion classification ($E_C$):

According to the results presented in Table 10, we can observe that the performance of the emotion task increases as we transition from single-task learning to dual-task and eventually to tri-task learning. In the single-task setup, the CM-RFT model outperforms the CM-T and CM-FT models across all three feature combinations, indicating that incorporating sentiment and emoji information can enhance the emotion detection task’s performance. In the dual-task setup with emoji, the performance of all models is considerably lower than in the single-task setup. However, the performance improves as more features are incorporated, and the CM-RFT model achieves the best results with all three features. This suggests that utilizing various feature types can benefit joint learning of emoji and emotion detection, and the tri-task setup may provide further improvement. In the dual-task setup with the sentiment, the performance is better than with emoji. The addition of Elmo embeddings and TF-IDF features leads to consistent performance improvement, with the CM-RFT model again achieving the best results. This implies that joint learning of sentiment and emotion detection can also benefit from the use of multiple feature types.

The presence of sentiment and emoji information appears to enhance the emotion task’s performance, as suggested by the results. The best performance for the emotion task was obtained in the tri-task learning setup, which involved jointly learning sentiment, emotion, and emoji detection tasks. The improvement in performance can be attributed to the fact that sentiment and emoji provide additional contextual information that can help in better disambiguation of emotions.

The results also suggest that multitask learning is more effective than single-task learning, especially when the tasks are related, such as emotion, sentiment, and emoji detection. The emotion task’s performance improved consistently as we progressed from single-task to dual-task and finally to tri-task learning. This indicates that joint learning of related tasks can better utilize the available information and improve the overall performance of the system.

### Comparison with existing works

The presented results in Table 11 indicate that the *CM-RFT* model proposed in this study performs better than the state-of-the-art models for both sentiment and emoji detection tasks. In the single-task scenario, mBERT achieved the highest accuracy of 63.77% and an F1 score of 61.54% for the emoji detection task. However, in the multi-task setting, the proposed *CM-RFT* model surpasses all other models, achieving an accuracy of 75.81% and an F1 score of 73.25%. This shows that the proposed model effectively uses multi-task learning to improve the performance of both tasks. Moreover, the model also shows promising results for the unsupervised emotion detection task, with an F1 score of 60.53% and an accuracy of 63.73%. This demonstrates that the zero-shot approach utilized in the proposed model is effective in detecting emotions from the text even without labeled data.

When focusing on the emoji prediction task, the proposed *CM-RFT* model outperforms both single-task and multi-task models significantly. The model achieves an accuracy of 75.81%, which is approximately 12% higher than the accuracy of the best-performing single-task model (mBERT) and approximately 9% higher than the accuracy of the best-performing multi-task model (TL-XLMR$^{[LS]}$). Moreover, the model’s F1 score is 73.25%, which is approximately 12% higher than the F1 score of the best-performing single-task model (mBERT) and approximately 8% higher than the F1 score of the best-performing multi-task model (TL-XLMR$^{[LS]]}$).

We conducted additional experiments with our proposed model to compare it fairly with the single- and multi-task baselines discussed earlier. As none of the baseline models addressed unsupervised classification, they couldn’t generate scores for the emotion task, unlike our proposed *CM-RFT* model that solves sentiment and multi-label emoji detection in a supervised setting and emotion detection in an unsupervised setting using a zero-shot approach. Therefore, we trained two versions of the *CM-RFT* model: one in a single-task setting (*CM-RFT*$^{STL}$ $_{[-Emo]}$) for all tasks and another in a multitask setting (*CM-RFT*$^{MTL}$ $_{[-Emo]}$) without the emotion task. The results are presented in Table 11.

Comparing the performance of *CM-RFT*$^{STL}$ $_{[-Emo]}$ with single-task models XLMR, XLMR$^{[FT+LS+RF]}$, mBERT, we observe that STL-CM-RFT outperforms all these models in terms of accuracy and F1 scores for the emoji and sentiment tasks. For example, the accuracy of *CM-RFT*$^{STL}$ $_{[-Emo]}$ is 67.30% for the emoji task, while the highest accuracy achieved by single-task models is 63.77% by mBERT. Similarly, *CM-RFT*$^{STL}$ $_{[-Emo]}$ achieves an F1 score of 74.64% for sentiment detection, while the highest F1 score achieved by single-task models is 70.32% by mBERT. These results indicate that the inclusion of the unsupervised emotion task has indeed helped the model perform better on supervised tasks.

Comparing the performance of *CM-RFT*$^{MTL}$ $_{[-Emo]}$ with multi-task models MT-XLMR, TL-XLMR$^{[LS]}$, TL-mBERT[*LS*], we observe that *CM-RFT*$^{MTL}$ $_{[-Emo]}$ outperforms all these models in terms of accuracy and F1 scores for both emoji and sentiment tasks. For example, the accuracy of *CM-RFT*$^{MTL}$ $_{[-Emo]}$ is 71.68% for the emoji task, while the highest accuracy achieved by multi-task models is 66.83% by TL-XLMR$^{[LS]}$. Similarly, *MT-CM-RFT* achieves an F1 score of 78.22% for sentiment detection, while the highest F1 score achieved by multi-task models is 72.58% by MT-XLMR. These results indicate that the inclusion of the unsupervised emotion task has indeed helped the model perform better in both single-task and multi-task settings.

We evaluate the performance of Llama model on the emotion recognition task by fine-tuning it for three epochs. Our model yielded an F1 score of 60.53 for emotion recognition which positions closely alongside the Llama model, which achieved an F1 score of 61.11. These results underscore the effectiveness of our proposed approach in tackling emotion recognition tasks, indicating its potential for practical applications in natural language processing.

To sum up, the *CM-RFT* model we proposed outperforms the current state-of-the-art models in both sentiment and emoji detection tasks. Our results indicate that taking advantage of multi-task learning and utilizing a zero-shot approach for unsupervised emotion detection can lead to substantial improvements in task performance. For the emoji prediction task, our proposed model achieves a remarkable improvement over the best-performing single-task and multi-task models, demonstrating the efficacy of our approach.

**Table 11:** Comparisons between our proposed model and the other baselines. The maximum scores are displayed in bold. Results marked with a * are benchmark results for the proposed task.

Models	Emoji		Sentiment		Emotion	
	F1 (%)	A (%)	F1 (%)	A (%)	F1 (%)	A (%)
Singletask baselines						
*XLMR*	58.18	61.21	67.42	70.38	-	-
$\textit{XLMR}^{[FT +LS+RF]}$^86^	59.89	61.04	68.55	71.43	-	-
$\textit{mBERT}$^87^	61.54	63.77	70.32	73.21	-	-
CM-RFT$^{STL*}$ $_{[-Emo]}$ (proposed)	*64.14*	*67.30*	*74.64*	*77.31*	*55.76*	*58.63*
Multitask-baselines						
$\textit{XLMR}^{MTL}$^87^	63.52	64.88	72.58	75.44	-	-
$\textit{TL-XLMR}_{[LS]}$^6^	65.30	66.83	78.21	80.55	-	-
$\textit{TL-mBERT}_{[LS]}$^6^	64.68	65.77	76.59	78.23	-	-
CM-RFT$^{MTL*}$ $_{[-Emo]}$ (proposed)	*68.57*	*71.68*	*78.22*	*79.21*	-	-
Multitask-Zeroshot-Emotion-BenchMark						
CM-RFT* (proposed)	73.25	75.81	83.14	82.35	60.53	63.73

### Comparison with current models across various datasets

To assess the effectiveness of our model, we conducted comparisons with several papers and their corresponding models.

- Comparison Study 1: “Emotion Detection in Code-Mixed Roman Urdu - English Text”^51^. Models: We compared our model with BERT and XLM-RoBERTa. Dataset Used: We used the Code-Mixed Roman Urdu - English Text dataset. The results, as shown in Table 12, indicate that our model outperforms both BERT and XLM-RoBERTa with an F1 score of 0.69, demonstrating its effectiveness in detecting emotions in code-mixed text.
- Comparison Study 2: “A self-attention hybrid emoji prediction model for code-mixed language”^92^ Models: We compared our model with BARF. Dataset Used: We used the Hinglish Emoji Prediction (HEP) dataset. The results, as presented in Table13, indicate that our model achieves a higher F1 score of 0.64 compared to BARF, demonstrating its superior performance in predicting emojis in code-mixed language.
- Comparison Study 3: “Multitasking of sentiment detection and emotion recognition in code-mixed Hinglish data”^6^ Models: We compared our model with TL-XLMR$^{MTL}_{LS}$. Dataset Used: We used the SemEval-2020 Task 9 dataset^93^. Table14 displays the results, showing that our model achieves higher F1 scores for both emotion detection (76.22) and sentiment analysis (70.31) compared to TL-XLMR$^{MTL}_{LS}$, indicating its effectiveness in multitasking for sentiment and emotion recognition in code-mixed Hinglish data.

**Table 12:** Comparison on Code-Mixed Roman Urdu - English Text dataset.

Model	F1 Score(Emotion)
BERT	0.28
XLM-Roberta	0.60
$CM-RFT^{STL}$	0.69

**Table 13:** Comparison on Hinglish Emoji Prediction (HEP) dataset.

Model	F1 Score(Emoji)
BARF	0.59
$CM-RFT^{STL}$	0.64

**Table 14:** Comparison on SemEval-2020 Task 9 dataset.

Model	F1 Score (Emotion)	F1 Score (Sentiment)
TL-XLMR$^{MTL}_{LS}$	71.61	64.47
$CM-RFT^{MTL}$	76.22	70.31

### Ablation study

#### Ablation experiments on the components of the proposed *CM-RFT* framework

Table 15 shows the results of four ablation experiments aimed at evaluating the contribution of different components in the proposed *CM-RFT* framework. The four components examined are the GLU module, the auto-encoder and ANP module, the self-attention mechanism, and the collective combination of GLU, self-attention, ANP, and AE modules.

The results indicate that each component contributes to the overall performance of the *CM-RFT* framework. Removing any of these components leads to a significant decline in F1 scores for all three tasks, especially when all four modules are removed (row 4). This suggests that the proposed framework is well-designed, and each module plays a critical role in its success. Specifically, the GLU module seems to be a crucial part of the framework (row 1). The removal of this component leads to a significant decrease in performance across all three tasks, highlighting the importance of non-linear transformations in the text encoder. Similarly, removing the auto-encoder and ANP module leads to a drop in performance (row 2), indicating the importance of these unsupervised pre-training methods in learning useful feature representations. Moreover, the self-attention mechanism appears to be more effective than linear concatenation in fusing the output features of the GLU and Trans Encoder modules (row 3). This result confirms the superior performance of self-attention in capturing long-range dependencies and modeling interactions among input tokens. Finally, the collective combination of GLU, SA, ANP, and AE modules is a highly effective feature learning mechanism (row 4), but it also leads to higher computational costs. The result suggests that one can still achieve decent performance with a simpler linear concatenation mechanism, albeit at the cost of reduced model capacity and expressive power.

In summary, the ablation experiments demonstrate the importance of each module in the proposed *CM-RFT* framework for multi-label emoji prediction. The findings can guide the design of future models and shed light on the underlying mechanisms that contribute to their success.

**Table 15:** Ablation experiment’s results. % fall in scores are shown in brackets. GLU: Gated Linear Unit, SA: Self-attention fusion, AE: Auto-encoder, ANP: Adjective-Noun Pair.

Setup	F1$^{Emoji}$ (%)	F1$^{Emotion}$ (%)	F1$^{Sentiment}$(%)
*[CM-RFT]*$_{-GLU}$	69.97 ($-3.79$)	57.39 ($-3.41$)	78.54 ($-3.81$)
*[CM-RFT]*${-(AE+ANP)}$	70.97 ($-2.79$)	58.15 ($-2.38$)	79.34 ($-3.01$)
*[CM-RFT]*${-SA}$	71.78 ($-1.98$)	58.22 ($-2.31$)	79.69 ($-2.66$)
*[CM-RFT]*${-(GLU+SA+AE+ANP)}$	66.97 ($-6.79$)	53.52 ($-7.01$)	75.37 ($-6.98$)
*[CM-RFT]*	**73.76**	**60.53**	**82.35**

**Table 16:** Effect of different loss functions on the model. Here, the Basic Model defines our basic structure where semantic features are directly combined via zero-shot loss function. The maximum scores are displayed in bold.

Basic Model	${\mathcal {L}}_{ad}$	${\mathcal {L}}_{re}$	${\mathcal {L}}_{al}$	$F1^{Emoji}$ (%)	$F1^{Emotion}$ (%)	$F1^{Sentiment}$ (%)
✓	–	–	–	69.60 (−4.16)	56.09 (−4.44)	78.08(−4.27)
✓	✓	–	✓	72.25 (−1.51)	58.96 (−1.57)	80.52(−1.83)
✓	–	✓	✓	71.73 (−2.03)	58.74 (−1.79)	80.14(−2.21)
✓	–	–	✓	70.77 (−2.99)	57.22 (−3.31)	78.23(−4.12)
✓	✓	✓	✓	**73.76**	**60.53**	**82.35**

Significant values are in bold.

#### Importance of loss functions for model training

Table 16 shows the results of four ablation experiments where each experiment is compared to the proposed *CM-RFT* containing all three loss functions (${\mathcal {L}}_{ad}$, ${\mathcal {L}}_{re}$, and ${\mathcal {L}}_{al}$) for the emoji, emotion, and sentiment tasks.

The F1 scores for all three tasks consistently decrease in each ablation experiment when any of the loss functions are removed. The largest decrease in performance is observed when all three loss functions are removed, indicating that each loss function plays an important role in the model’s performance. Specifically, removing the ${\mathcal {L}}_{ad}$ and ${\mathcal {L}}_{re}$ loss functions has the greatest negative impact on the model’s performance compared to removing only one of these loss functions. This suggests that these loss functions contribute significantly to the model’s ability to capture relevant features for both the adversarial training and reconstruction of the input data.

In terms of the contributions of the individual loss functions, the adversarial loss (${\mathcal {L}}_{ad}$) appears to have a slightly larger impact on performance compared to the alignment loss (${\mathcal {L}}_{al}$) and reconstruction loss (${\mathcal {L}}_{re}$), especially for the emoji and emotion detection tasks. This indicates that adversarial loss plays an important role in the model’s ability to distinguish between different classes for these tasks. On the other hand, the alignment loss and reconstruction loss appear to be more important for sentiment detection.

Overall, these results demonstrate the importance of the proposed loss functions for effective training of the multitask emoji, emotion, and sentiment detection system. These findings can be used to guide the development of more effective training strategies for multitasking learning models in the future. For example, incorporating additional loss functions or modifying the weighting of existing loss functions may improve the model’s performance. Additionally, these results suggest that the importance of different loss functions may vary depending on the specific tasks being performed and the data being used, highlighting the importance of careful analysis and selection of loss functions in the design of multitask learning models.

**Table 17:** The proposed framework’s predictions for Emoji, Emotion, and Sentiment are shown below.

Single label Emoji prediction							
Code-Mixed Tweet	English Translation	Actual			Predicted		
		Emoji	Emotion	Sentiment	Emoji	Emotion	Sentiment
I don’t know tu itna acha kaise hai bhai aaj bhi	I don’t know how you are too good to even today		Joy	Positive		Joy	Positive
tere ghamand k karan hi aaj congress k ye halat hai . failure hai tu Bhai .. Tujhse na ho payega	Because of your pride, this is the condition of Congress today. .. you are failure..you cant do this		Anger	Negative		Disgust	Negative
Congress ki sarker mai cylinder he gayab ho gaya tha	During congress govt. cylinder went missing		Disgust	Negative		Disgust	Negative
Multilabel emoji							
Code-Mixed Tweet	English Translation	Actual			Predicted		
		Emoji	Emotion	Sentiment	Emoji	Emotion	Sentiment
meta RT @ narendramodi Congratulations sir ji Dobara pm banee ki hardik subhkamnaye aapko	meta RT @ narendramodi Congratulations sir to become PM for the second time.	, ,	Joy	Positive	,	Joy	Positive
meta RT @ Chaosthestic Secondly maa baap ki izzat b koi cheez hoti hai. Har cheez ko support karna band karo . Just because she is a woman doesnt mean wo kch bhi bolegi	meta RT @ Chaosthestic Secondly father and mother respect is the something. dont support everything. Just because she is a woman doesnt mean she can say anything.	, ,	Anger	Negative	, ,	Disgust	Negative
I really wanna meet him to show my love with my hand.. saale ne jeena muskil kar rakha hai.	I really wanna meet him to show my love with my hands .. he made my life miserable.	, ,	Sad	Negative	,	Anger	Negative

### Qualitative analysis

In this section, we provide a qualitative analysis of our proposed multitask framework, which takes into account the relationship between emoji, sentiment, and emotion, as we previously mentioned. To illustrate the impact of these tasks on one another, we have selected several examples from the *SENTIMOJI* dataset and present them in Table 17.

- *Observation 1:* In the first sentence, the model correctly predicts a heart emoji, positive sentiment, and joy as the emotion. The model seems to have picked up on the positive sentiment and joy from the words “too good” and “don’t know” respectively, and predicted the heart emoji to match the positive sentiment. Moreover, the word “bhai” (brother) may imply a friendly or affectionate tone, leading to the identification of the heart emoji. Finally, the presence of the word “joy” or similar words in the training data might have helped the model to identify the emotion accurately.
- *Observation 2:* In the second sentence, the model correctly predicts the negative sentiment, but the predicted emoji is wrong. The model predicted a pouting face instead of an angry face, which could be because the pouting face emoji can also indicate dissatisfaction or annoyance, which might be related to pride. Additionally, the emotion is misclassified as disgust instead of anger, which could be because of the strong negative sentiment and the use of words like “failure” and “can’t do this”.
- *Observation 3:* In the third sentence, the model correctly predicts the Face With Open Mouth, Throwing Up emoji, indicating disgust, along with the negative sentiment. The sentence contains words like “missing,” which suggests a negative sentiment, and the use of the Face With Open Mouth, Throwing Up emoji, and disgust emotion can be related to the revulsion expressed in the sentence.
- *Observation 4:* In the first multi-label sentence, the model correctly predicts the negative sentiment and joy as the emotion, but only partially predicts the emojis. The use of “hardik subhkamnaye” and “Congratulations sir ji” in the sentence indicates a positive sentiment and the use of “Dobara pm banee” suggests a sense of achievement, which could explain the use of the heart and sparkles emojis. The misclassification of the smiling face emoji could be due to the lack of contextual information or insufficient training data.
- *Observation 5:* In the second multi-label sentence, the model correctly predicts the negative sentiment but misclassifies the emotion as disgust instead of anger. For the emojis, the model predicted pouting face, crying face, and dissapointed face, but the original annotations have pouting face, angry face, and Face With Open Mouth, Throwing Up. This could be because the model picked up on the negative sentiment and the use of words like “respect”, “anything”, and “woman”, which might have led to the prediction of the pouting face emoji, while the crying face and dissapointed face emojis could be related to the negative sentiment.
- *Observation 6:* In the third multi-label sentence, the model correctly identifies the sentiment as negative but wrongly predicts the emotion as anger instead of sad. The model also partially predicts the emojis, which may be due to the presence of multiple emotions in the sentence. To improve the prediction, the model could be trained on more data that contains similar phrases and words to better distinguish between different negative emotions and emojis.

### Error analysis

The analysis of the incorrect predictions revealed several common error patterns, which are summarized below:

1. Ambiguity in Emoji Interpretation: The model often struggles with emojis that have multiple interpretations depending on the context. For example, the emoji can represent both laughter and tears of joy, leading to misclassifications.
2. Negation and Sarcasm: Negation and sarcasm in text can lead to misinterpretations by the model, especially in sentiment analysis. For instance, the phrase “not bad” may be interpreted as positive by the model, leading to misclassification.
3. Lack of Context: The model sometimes fails to capture the context of a sentence, leading to errors in sentiment and emotion classification. For example, short or contextually ambiguous sentences may be misclassified.
4. Data Imbalance: Imbalance in the distribution of classes can lead to biases in the model’s predictions, especially for minority classes. This is particularly evident in emotion classification, where some classes have fewer examples than others.
5. Out-of-Vocabulary Words: The presence of out-of-vocabulary words in the text can lead to errors, especially when the model is unable to capture their semantics. This is more common in emoji and sentiment analysis tasks.

These error patterns highlight the challenges faced by the proposed CM-RFT model in understanding and interpreting text across different tasks. Addressing these challenges requires further research into more robust modeling techniques, better handling of context and ambiguity, and mitigation of biases in the data.

The joint learning of sentiment and emotion tasks with the emoji prediction task may have benefited the performance of the emoji task. This is because emotions and sentiments can provide additional context for the model to predict the appropriate emojis. For example, in the first correct prediction sample, the model was able to correctly predict the heart emoji, which may have been influenced by the positive sentiment and joyful emotion predicted for the sentence. Similarly, in the second incorrect prediction sample, the model correctly predicted the negative sentiment but misclassified the emotion and emoji, suggesting that it may not have fully captured the nuances of the text.

Single-label emojis can be a risk in multilabel emoji prediction because the emojis can have different meanings in different contexts, and a single emoji may not be able to capture all the nuances of the text. For example, the “pouting face” emoji can be used to express anger, disappointment, or sadness, and without additional context, it can be difficult to determine the exact emotion being conveyed. We observe in the incorrect prediction samples, that the model has predicted some of the emojis correctly while missing some. It is better than having fully incorrect predictions because it shows that the model has some understanding of the context and can predict the relevant emojis to some extent. However, there is still room for improvement in the model’s performance.

To improve the model’s predictions, we can consider the following steps:

- *Increase the training data:* The model might benefit from additional training data to capture the various nuances of language and emotions.
- *Incorporate context:* The model might benefit from incorporating the context of the sentence to better identify the sentiment, emoji, and emotion.
- *Use pre-trained language models:* The model might benefit from using pre-trained language models that can capture the semantic meaning of words and phrases.
- *Regularize the model:* The model might benefit from regularization techniques to prevent overfitting and improve generalization.
- *Analyze and correct errors:* Analyzing the model’s errors and correcting them might help improve the model’s performance over time.

### Comparison with ChatGPT-3.5

We perform a study using ChatGPT(https://chat.openai.com/) to demonstrate the effectiveness of our proposed framework. We notice that **CM-RFT** has an overwhelming performance advantage over ChatGPT. A few sample predictions from ChatGPT on the *TASKS* task are shown below:

Prompt: *Read these hinglish utterances and find the suitable emojis, emotion, and sentiment:*

- tere liye chand nhi la sakta baby actually tu bhaad mein ja
- Tere ghamand k karan hi aaj congress k ye halat hai ... failure hai tu Bhai .. Tujhse na ho payega
- Congress ki sarker mai cylinder he gayab ho gaya tha
  - Human Annotators:
  - Emoji Label: , ,
  - Emotion Label: Anger, Anger, Disgust.
  - Sentiment Label: Negative, Negative, Negative
  - Proposed_MODEL:
  - Emoji Label: , ,
  - Emotion Label: Anger, Disgust, Disgust.
  - Sentiment Label: Negative, Negative, Negative
  - ChatGPT:
  - Emoji Label: , ,
  - Emotion Label: Dismissive, Anger, Confusion.
  - Sentiment Label: Negative, Negative, Neutral (depending on the context, it could be interpreted as negative)

In our analysis, it is evident that our model yields results akin to ChatGPT. While ChatGPT is renowned for its high performance, our model demonstrates proficiency, particularly in handling codemixed sentences.

### Limitations and potential biases

While our proposed CM-RFT model demonstrates strong performance across multiple tasks, there are several limitations and potential biases that need to be addressed:

1. Data Bias: The performance of the model heavily relies on the quality and representativeness of the training data. Biases present in the training data, such as underrepresentation of certain demographics or topics, can lead to biased predictions by the model.
2. Language Bias: The model’s performance may vary across different languages due to differences in linguistic structures, cultural nuances, and availability of training data. It may perform better on languages that are well-represented in the training data compared to those that are not.
3. Context Sensitivity: The model’s performance is influenced by the context in which the text is presented. It may struggle with contextually ambiguous or sarcastic text, leading to misinterpretations.
4. Generalization: The model’s ability to generalize to unseen data or domains is limited by the diversity and representativeness of the training data. It may perform well on data similar to the training data but struggle with out-of-domain or adversarial examples.
5. Interpretability: The complex architecture of the proposed *CM-RFT* model may hinder its interpretability, making it challenging to understand how and why certain predictions are made. This lack of interpretability can limit the model’s usefulness in real-world applications where transparency and accountability are important.

Addressing these limitations and biases requires careful consideration of model design, training data, evaluation metrics, and ethical considerations. Future research should focus on developing more robust and fair AI models that are capable of handling diverse languages, cultures, and contexts while ensuring transparency, interpretability, and accountability. Additionally, efforts should be made to collect more diverse and representative training data and to develop evaluation metrics that account for biases and fairness concerns. By addressing these challenges, we can build AI models that are more reliable, equitable, and trustworthy for real-world applications.

## Conclusion

In conclusion, our research proposes a novel task of predicting multiple emojis in code-mixed sentences while identifying the corresponding sentiment and emotion of the user. We have introduced a zero-shot strategy to integrate emotional information into the training process and developed an end-to-end framework to identify sentiment, emotion, and corresponding emojis simultaneously. We have also introduced the SENTIMOJI dataset by extending the benchmark sentiment annotated SentiMix Dataset of code-mixed tweets with manual annotations for multi-label emojis. Our proposed multi-task framework can help bridge the research gaps in the field of sentiment and emotion analysis by providing a comprehensive understanding of the relationship between emojis, sentiment, and emotion in code-mixed sentences. Our work lays the foundation for future studies to improve natural language processing tasks that require a deeper understanding of code-mixed text’s complex interdependence between language, sentiment, and emojis. The proposed approach can be extended to other languages and domains and can be integrated into social media platforms to improve emoji prediction and sentiment analysis for code-mixed text.

One limitation of our work is that we did not explore the use of more advanced pre-training techniques such as self-supervised learning. By incorporating such techniques into our model, we can enhance the way the model learns representations and potentially improve its performance. Although we achieved state-of-the-art results, there is still room for improvement by exploring more intricate architectures and further fine-tuning hyperparameters. In future research, the effectiveness of our proposed method can be tested on other code-mixed datasets in different languages to evaluate its generalization ability. It would be fascinating to investigate how our model’s transferability to other languages performs in low-resource settings. Finally, our approach can be extended to other NLP tasks, such as named entity recognition, machine translation, and speech recognition, in code-mixed languages.

## Data Availability

This study involves working with the publicly available SentiMix dataset (https://zenodo.org/record/3974927#.ZEva4pFBxH6) and the authors do not violate any copyright norms. The codes and data will be made available at https://www.iitp.ac.in/~ai-nlp-ml/resources.html#SENTIMOJI
